# Prenatal exposure to alcohol and its impact on reward processing and substance use in adulthood

**DOI:** 10.1038/s41398-024-02941-9

**Published:** 2024-05-28

**Authors:** Klara Mareckova, Radek Marecek, Lenka Andryskova, Milan Brazdil, Yuliya S. Nikolova

**Affiliations:** 1 Central European Institute of Technology (CEITEC), Masaryk University, Brno, Czech Republic; 2 First Department of Neurology, Faculty of Medicine, Masaryk University and St. Anne’s University Hospital, Brno, Czech Republic; 3 RECETOX, Masaryk University, Brno, Czech Republic; 4 Campbell Family Mental Health Research Institute, Centre for Addiction and Mental Health (CAMH), Toronto, ON Canada; 5 Department of Psychiatry, University of Toronto, Toronto, ON Canada

**Keywords:** Pathogenesis, Neuroscience

## Abstract

Heavy maternal alcohol drinking during pregnancy has been associated with altered neurodevelopment in the child but the effects of low-dose alcohol drinking are less clear and any potential safe level of alcohol use during pregnancy is not known. We evaluated the effects of prenatal alcohol on reward-related behavior and substance use in young adulthood and the potential sex differences therein. Participants were members of the European Longitudinal Study of Pregnancy and Childhood (ELSPAC) prenatal birth cohort who participated in its neuroimaging follow-up in young adulthood. A total of 191 participants (28–30 years; 51% men) had complete data on prenatal exposure to alcohol, current substance use, and fMRI data from young adulthood. Maternal alcohol drinking was assessed during mid-pregnancy and pre-conception. Brain response to reward anticipation and reward feedback was measured using the Monetary Incentive Delay task and substance use in young adulthood was assessed using a self-report questionnaire. We showed that even a moderate exposure to alcohol in mid-pregnancy but not pre-conception was associated with robust effects on brain response to reward feedback (six frontal, one parietal, one temporal, and one occipital cluster) and with greater cannabis use in both men and women 30 years later. Moreover, mid-pregnancy but not pre-conception exposure to alcohol was associated with greater cannabis use in young adulthood and these effects were independent of maternal education and maternal depression during pregnancy. Further, the extent of cannabis use in the late 20 s was predicted by the brain response to reward feedback in three out of the nine prenatal alcohol-related clusters and these effects were independent of current alcohol use. Sex differences in the brain response to reward outcome emerged only during the no loss vs. loss contrast. Young adult men exposed to alcohol prenatally had significantly larger brain response to no loss vs. loss in the putamen and occipital region than women exposed to prenatal alcohol. Therefore, we conclude that even moderate exposure to alcohol prenatally has long-lasting effects on brain function during reward processing and risk of cannabis use in young adulthood.

## Introduction

Despite the recommended abstinence from alcohol during pregnancy, alcohol use during this critical period for child’s brain development remains common. A large multinational European study reported that the prevalence of alcohol use during pregnancy was 28.5% in the United Kingdom, 26.5% in Russia, and 20.9% in Switzerland [1]. Data from the National Birth Defects Prevention Study in the United States then showed that some alcohol drinking during pregnancy has been reported by 30% of women, decreasing to 22% after the first month and to 8% during the third trimester [2].

Heavy maternal alcohol drinking during pregnancy (e.g. 7 or more drinks per week according to Flak et al. [3], which based on the WHO definition of a standard drink means approximately 70 g of alcohol per week or more [4]) is known to be linked with cognitive difficulties, worse attention, motor deficits, or memory and language problems in the offspring [5–8]. This range of behavioral deficits in children with confirmed heavy prenatal alcohol exposure is referred to as the fetal alcohol spectrum disorder (FASD). Consistently with these behavioral deficits, functional magnetic resonance imaging (fMRI) studies of people with FASD versus healthy controls showed atypical brain response during inhibitory control [9], verbal learning [10], and verbal [11] as well as visual [12] and spatial [13] working memory. Most of these studies reported higher brain response in dorsal frontal cortices, which are involved in executive functions, in children with FASD regardless of the fMRI task [9–11, 13]. Maternal alcohol drinking during pregnancy has been linked also with smaller brain volumes, thinner cortex [14], and altered white matter microstructure [15–19].

Further research reported a negative impact of lighter alcohol drinking (6 or less drinks per week [3]) during pregnancy on behavioral outcomes. Using data from the ABCD study, Lees et al. [20] conducted the largest examination of prenatal alcohol exposure on neurodevelopmental outcomes in preadolescence and showed that prenatal alcohol exposure of any severity was associated with greater psychopathology, attention deficits, and impulsiveness. However, a recent review of 36 studies regarding low to moderate prenatal alcohol exposure and its behavioral and cognitive outcomes [21] concluded that the results are inconsistent, most likely due to methodological issues such as retrospective biases, heterogeneity of exposure definitions, lack of time specificity or confounding variables. Thus, prospective longitudinal studies are needed to clarify the link between prenatal exposure to alcohol and brain development.

Maternal alcohol drinking during pregnancy is also known as one of the best predictors of later alcohol use in adolescence and young adulthood [22, 23]. The absence of such a relationship was reported only by two studies in children [24] and adolescents [25]. Preclinical research [26] reported that male rats exposed to alcohol prenatally showed reduced goal-directed sucrose self-administration, suggesting reduced motivation and greater anhedonia. Such lack of motivation is also known to be associated with high risk of compulsivity and impulsivity, which are both highly related to obesity and substance abuse [26]. Further research in monkeys also demonstrated effects of prenatal alcohol on the vulnerability of the dopamine system and pointed out that continuously exposed monkeys showed the largest effects [27]. According to a preclinical study by Hausknecht et al. [28], the increased risk for drug addiction in the offspring exposed to alcohol prenatally can be explained by decreased function of endocannabinoid receptors, which weaken the excitatory synaptic strength in dopamine neurons in the brain. Consistently, further research reported the effects of prenatal alcohol exposure on endocannabinoid function [29] as well as the development of the dopaminergic system [30, 31], inducing sensitivity to the stimulant effects of alcohol [32, 33]. Given these preclinical findings as well as further preclinical research demonstrating that prenatal exposure can sensitize the offspring to the effects of alcohol and drugs and increase their preference for alcohol and drugs [34–36], it is possible that similar sensitization after the prenatal alcohol exposure might also occur in humans.

While the impact of heavy alcohol drinking during pregnancy on child’s neurodevelopment has been reported by numerous studies, the effects of low-dose alcohol drinking are less clear, and any potential safe level of alcohol use during pregnancy is not known. As reported by Dodge et al. [37], it is also difficult to tease out to what extent is substance use in adulthood determined by the exposure to alcohol during pregnancy in particular versus other potentially confounding factors associated with alcohol drinking pre-conception. Given the effects of prenatal alcohol exposure on endocannabinoid function [28, 29] and dopamine signaling [30, 31], we hypothesize that exposure to maternal alcohol use during pregnancy might be associated not only with higher alcohol use in the offspring but also propensity to higher cannabis use as well as broader alterations in neural reward processing. Given the effects of prenatal alcohol on social behavior, learning, memory, and behavioral flexibility [38], prenatal alcohol exposure may have strongest effects on the somatosensory area, amygdala, hippocampus, striatum, and prefrontal cortex. Finally, given the literature on striking sex differences in the response to early-life alcohol exposure [39] as well as higher neural sensitivity to reward in men vs. women [40], we also hypothesize that the effects of prenatal alcohol on reward-related behavior and cannabis use might differ between men and women. To answer these questions, we have conducted a neuroimaging follow-up of the European Longitudinal Study of Pregnancy and Childhood (ELSPAC) prenatal birth cohort in young adulthood and assessed the relationships between maternal alcohol use during pre-conception and mid-pregnancy and substance use and monetary reward processing in the offspring in their late 20 s.

## Materials and methods

### Participants

All participants were members of the ELSPAC [41, 42] prenatal birth cohort and thus born in South of Moravia, the Czech Republic, between 1991 and 1992. All of them were 28–30 years when participating in the neuroimaging follow-up focused on reward processing and substance use. All participants provided written informed consent to participate in the neuroimaging follow-up (Health Brain Age study), including the agreement to merge these newly collected data with their historical data from ELSPAC. Ethical approval for the Health Brain Age study was obtained from the ELSPAC ethics committee. The current study also followed the Strengthening the Reporting of Observational Studies in Epidemiology (STROBE) reporting guideline for cohort studies.

### Procedures

Alcohol drinking during preconception and mid-pregnancy were assessed during the 20th week of pregnancy using a self-report questionnaire. The self-report questionnaires during the 20^th^ week of pregnancy also assessed maternal education and maternal depression during pregnancy, the latter with the Edinburgh Postnatal Depression Scale (EPDS).

Structural and functional magnetic resonance imaging (MRI) was acquired using a 3 T Siemens Prisma MRI Scanner. Reward processing in the young adult offspring was tested using a Monetary Incentive Delay (MID) fMRI task [43, 44]. Substance use was assessed on the same day using a self-report questionnaire, which asked the young adult (1) How many times in the past 30 days did you drink (a) beer, (b) wine, (c) shots, (2) How many times in the past 30 days did you smoke cigarettes, and (3) How many times in your life did you try cannabis. The alcohol use in the past month as well as the lifetime cannabis use were reported as one of the following categories: 0, 1, 2, 3–5, 6–9, 10–19, 20–39, 40 and more. We coded these from 0 to 7, where 7 reflects the greatest alcohol or cannabis use. Cigarette smoking in the past month was reported as one of the following categories: none, less than once a week, less than once a day, 1–5 per day, 6–10 per day, 11–20 per day, more than 20 per day. While a total of 262 young adults participated in the neuroimaging follow-up and filled in questionnaires regarding substance use, 27% of them did not have complete information on the maternal alcohol drinking during pregnancy and thus could not be included in the current study. A total of 198 participants had complete information on current substance use and exposure to alcohol pre-conception, a total of 194 participants had complete information on current substance use and exposure to alcohol mid-pregnancy, and a total of 191 participants (49% women, 51% men) had complete data on prenatal exposure to alcohol, current substance use, as well as fMRI data from young adulthood. This sample size allowed us to detect with 5% error probability (p = 0.05) and 80% power medium (Cohen’s d = 0.538) or larger effects between the two groups.

### Acquisition of MRI and fMRI data

The fMRI data were collected using a multi-echo, multi-band echo planar imaging sequence (970 volumes ~11.5 min, TR = 0.7 s, 3 echos, TE1 = 16.4 ms, TE2 = 37.66, TE3 = 58.92, flip angle 47 deg, voxel dimensions: 3 × 3 × 3 mm). The field mapping data contained two gradient echo images acquired with different TE (4.92 ms and 7.38 ms) and the same resolution and orientation as fMRI data. The structural MRI data were acquired with a T1-weighted MPRAGE sequence.

### Monetary incentive delay (MID) fMRI task

As detailed in Dodge et al. [37], the MID task examines reward processing using monetary incentives. The MID task has good within-subject reliability over time [45] and is known for its association with addiction and addiction-related neural phenotypes [46–50]. Similarly to the ABCD study [43, 51], the MID task lasted approximately 12 min. It is a low cognitive demand task, instructing participants to respond to a target as quickly as possible. It models the anticipation of reward and receipt of reward as separate conditions, allowing to disentangle the motivational from the hedonic aspect of reward [43]. During the anticipation phase, the participant observes a cue signaling a reward (e.g. “Earn 100 CZK!“), loss (e.g. “Do not lose 100 CZK!”) or a neutral (“There is no money at stake”) trial, which is followed by a probe and the participant has to respond with a button press. The response is considered correct if participant press after probe onset and before the currently set time limit expires. In case of too early (i.e. before probe onset) or too late press the response is incorrect. During the receipt of reward phase, the participant receives performance feedback (e.g. “You pressed too slowly! You did not earn 100 CZK!” or “Correct response. You keep 100 CZK!”) and the time limit is adjusted according to response (Supplementary Table 1). There are 100 trials, 40/40/20 for reward/loss/neutral respectively. Each trial consisted of a cue image presented for 2 s, a fixation cross presented for 1–4 s (randomly), a probe image presented up to the currently set time limit or to the button press and feedback. The probe and feedback together lasted 2 s (for detail see Supplementary Fig. 1 and Supplementary Table 2). There were 12 variants of the task, each presenting the trials in a different order to avoid potential bias in neural activity due to order of reward, loss, and neutral trials. The specific variant was assigned to each participant at random.

### Pre-processing and analysis of fMRI data

The fMRI data were preprocessed by SPM12 toolbox (https://www.fil.ion.ucl.ac.uk/spm/) running under MATLAB R2017b (MathWorks, Inc.). All echo 2 data were realigned and resliced and echo 1 and 3 data were resliced using parameters from echo 2 data. Voxel displacement map was estimated according to the field mapping data and applied to all three-echo data to correct for geometrical distortions. Next, the three echo datasets were merged together using weighting by temporal signal-to-noise ratio. Subsequently, the mean fMRI image was registered to the structural image, which was spatially normalized to the MNI152 template. fMRI data were smoothed using FWHM of 5 mm and all preprocessed data were checked for subject motion. No subject had more than 20% of scans with a framewise displacement of 0.5 mm [52–54].

The SPM12 was used also for statistical analyses of fMRI data. The general lineal model (GLM) at the subject level contained 12 regressors that modeled responses to different aspects of stimulation: three regressors for cue interval (separately for reward, loss and neutral), three regressors for anticipation interval (separately for reward, loss and neutral), single regressor for probe, regressor for positive feedback during reward (earning some money), regressor for negative feedback during reward (earning no money), regressor for positive feedback during loss (losing no money), regressor for negative feedback during loss (losing some money) and single regressor for feedback during neutral. All regressors were constructed as convolution of boxcar function (onset and duration according to information logged during the running task) and canonical hemodynamic response function. The GLM was supplied by additional 12 regressors derived from movement parameters (actual values and temporal differences) estimated during fMRI data realignment to model effects of subject’s movement and by additional 10 regressors that modeled physiological noise at low frequencies (cut off 1/128 Hz). The GLM employed an autoregressive model to account for temporal autocorrelation in the fMRI time-series.

Voxelwise two-way ANOVA tested the effect of mid-pregnancy exposure to alcohol, participant sex, and their interaction on brain response during assessed by 4 subject-level contrasts: Anticipation of reward, Anticipation of neutral, Anticipation of loss, Reward feedback (vs. no reward feedback), and No loss feedback (vs. loss feedback). A similar model was also run for the exposure to alcohol pre-conception. Clusters that emerged by voxel-wise threshold at p < 0.001 (uncorrected) and that survived the threshold of FWEp < 0.05 were reported as significant. The effect size was calculated using Hedges’g for the main effect and using delta R^2^ for interaction terms calculated at the clusters’ T-value maxima. Finally, posthoc analyses evaluated the relationship between brain response to reward in the significant clusters and cannabis use in the late 20 s. In the posthoc analyses, multiple comparisons were corrected using a false discovery rate (FDR).

### Statistical analyses of the behavioral data

Upon checking the normal distribution of the data and the homogeneity of variance, a two-way ANOVA tested the effect of exposure to alcohol in mid-pregnancy, participant sex, and their interaction on (1) alcohol use in the past 30 days, (2) cigarette use in the past 30 days, and (3) lifetime cannabis use. A similar model was also used for the effect of exposure to alcohol preconception. Multiple comparisons were corrected using a false discovery rate (FDR).

## Results

### Demographics and prevalence of alcohol consumption in mid-pregnancy and pre-conception

Alcohol drinking was reported in 85% of women pre-conception and in 16% of women mid-pregnancy. Out of the 16% of women in mid-pregnancy, 3 reported daily alcohol drinking, 4 at least once a week and 25 less than once a week. Demographics table comparing the exposed and non-exposed groups showed that there were no differences in sex, age, ethnicity, BMI, education, anxiety or depressive symptoms in the offspring or maternal depression. The prenatally exposed vs. non-exposed groups differed only in the level of maternal education (Chi Square=12.22, p = 0.006; see Table 1 for details).

**Table 1:** Demographics table.

		Alcohol use in mid-pregnancy			Alcohol use pre-conception		
		Exposed (n = 33)	Non-exposed (n = 161)	Group difference	Exposed (n = 60)	Non-exposed (n = 138)	Group difference
Young adult offspring	Sex	19 F, 14 M	76 F, 85 M	Chi square = 1.18, p = 0.28	32 F, 28 M	64 F, 74 M	Chi square = 0.81, p = 0.37
	Age	M = 29.38, SD = 0.63	M = 29.41, SD = 0.57	t_(192)_ = −0.23, p = 0.82	M = 29.42, SD = 0.60	M = 29.39, SD = 0.57	t_(196)_ = 0.26, p = 0.79
	Ethnicity	100% European ancestry	100% European ancestry	No difference.	100% European ancestry	100% European ancestry	No difference.
	BMI	M = 25.52, SD = 5.91	M = 24.25, SD = 3.53	t_(192)_ = 1.65, p = 0.10	M = 24.50, SD = 5.09	M = 24.35, SD = 3.51	t_(196)_ = 0.24, p = 0.81
	Education in the late 20 s	0% elem. s. 24% high s. 7% undergrad 69% master 0% PhD	2% elem. s. 24% high s. 11% undergrad 62% master 1% PhD	Chi Square=1.84, p = 0.77	2% elem.s. 28% high s. 5% undergrad 65% master 0% PhD	1% elem.s. 22% high s. 12% undergrad 64% master 1% PhD	Chi Square=3.56, p = 0.47
	Depression (BDI score)	M = 8.03, SD = 8.19	M = 5.93, SD = 6.20	t_(192)_ = 1.51, p = 0.13	M = 6.52, SD = 6.02	M = 5.93, SD = 6.20	t_(196)_ = 0.92, p = 0.36
	Anxiety (STAI-T score)	M = 33.06, SD = 12.17	M = 30.33, SD = 9.28	t_(192)_ = 1.46, p = 0.15	M = 31.33, SD = 10.06	M = 30.64, SD = 9.88	t_(196)_ = 0.45, p = 0.65
Pregnant mother	Maternal education during pregnancy	16% elem. s. 25% high s. 59% university 0% postgrad.	22% elem. s. 47% high s. 28% university 3% postgrad.	**Chi Square** = **12.22, p** = **0.006**	19% elem. s. 35% high s. 41% university 5% postgrad.	21% elem. s. 48% high s. 29% university 2% postgrad.	Chi Square=4.86, p = 0.18
	Maternal depression during pregnancy (EPDS score)	M = 22.21, SD = 2.46	M = 21.69, SD = 2.35	t_(188)_ = 1.16, p = 0.25	M = 22.13, SD = 2.48	M = 21.58, SD = 2.33	t_(192)_ = 1.49, p = 0.14

### Prenatal alcohol and substance use in young adulthood

There was a significant interaction between prenatal alcohol and sex on alcohol use in the past month (beta = −0.18, p = 0.049; Adj R^2^ = 0.10) and post-hoc analyses revealed that while men showed greater alcohol use in the past month than women in both the exposed (t(31) = −4.33, p = 0.0001, Cohen’s d = 1.51) and the non-exposed (t(159) = −3.33, p = 0.001, Cohen’s d = 0.52) group, the sex difference was larger in the exposed group. While there was no significant effect of prenatal alcohol on cigarette smoking in the past month (beta = −0.02, p = 0.81), nor any interactions between prenatal alcohol and sex on cigarette smoking in the past month (beta = 0.04, p = 0.69), there was a significant effect of prenatal alcohol on lifetime cannabis use (beta = −0.19, p = 0.007, Adj R^2^ = 0.12), which did not interact with sex (beta = −0.07, p = 0.42). Only the effect of prenatal alcohol on cannabis use survived the FDR correction for multiple comparisons (alcohol: FDRp = 0.074, cigarettes: FDRp = 0.785, cannabis: FDRp = 0.021).

When correcting the model for maternal education and maternal depression during pregnancy, the effect of prenatal alcohol on lifetime cannabis use in young adulthood remained significant (beta = −0.18, p = 0.01, FDRp = 0.03, Adj R^2^ = 0.13) and the effects on alcohol and cigarette use in the past month remained insignificant (alcohol: beta = −0.06, p = 0.44; cigarettes: beta = −0.03, p = 0.68). There was also no interaction between prenatal alcohol and sex on the use of either substances (p > 0.08).

### Pre-conception exposure to alcohol and substance use in young adulthood

There was no significant effect of maternal alcohol drinking pre-conception on alcohol (beta = −0.12, p = 0.07) or cigarette smoking (beta = −0.03, p = 0.66) in the past month or lifetime cannabis use (beta = −0.04, p = 0.54) in the young adults. There were also no interactions between maternal alcohol drinking pre-conception and sex on either of the substance use variables (p > 0.12), supporting the possibility that the effects of prenatal exposure to alcohol on cannabis use are pregnancy-specific.

### Brain response to reward anticipation and reward feedback

Brain response to Anticipation of reward, Anticipation of loss, Reward feedback, and No loss feedback, which survived the initial voxel-wise uncorrected threshold of p < 0.001 as well as the cluster-level threshold of FWEp < 0.05 is described and illustrated in the Supplementary Results.

### Prenatal exposure to alcohol and reward processing in young adulthood

**Fig. 1:**
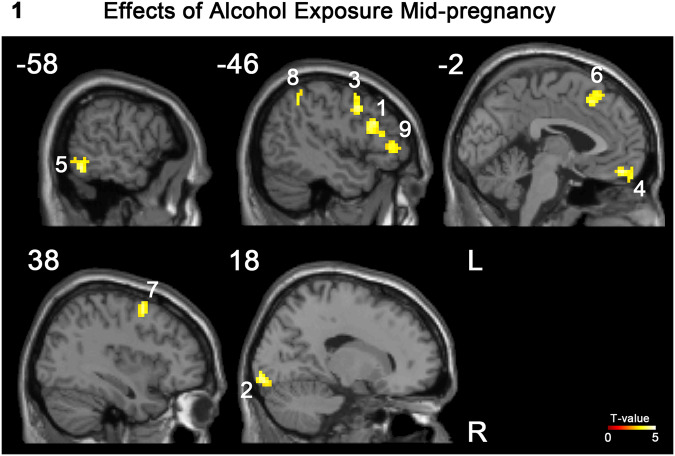
Prenatal exposure to alcohol and its impact on reward processing in young adulthood. Sagittal views of the 9 clusters showing significantly greater brain responses to reward in young adults prenatally exposed (vs. non-exposed) to alcohol.

Exposure to alcohol during mid-pregnancy was associated with reward feedback but not reward anticipation in the young adult offspring. Individuals exposed to alcohol prenatally showed greater brain response to reward feedback than individuals not exposed to alcohol prenatally in 9 clusters (see Fig. 1 and Table 2) including six frontal, one parietal, one temporal, and one occipital region, and these effects were independent of sex.

**Table 2:** Description of the 9 significant clusters showing greater brain response to reward in the young adults prenatally exposed to alcohol (independently of sex).

cluster		cluster – level				Number of voxels per AAL region
	Hedges’ g	p FWE	# voxels	T-value in maximum	x,y,z (mm)	
1	0.946	<0.001	85	5.21	−42 20 26	78 Frontal_Inf_Tri_L (aal); 1 Frontal_Inf_Oper_L (aal); 1 Frontal_Mid_L (aal)
2	0.878	0.002	63	4.79	15 −94 −10	46 Lingual_R (aal); 17 brodmann area 17; 7 Calcarine_R (aal); 3 Cerebelum_6_R (aal)
3	0.851	<0.001	140	4.73	−48 8 41	77 Frontal_Mid_L (aal); 59 Precentral_L (aal)
4	0.866	<0.001	103	4.65	−3 50 −19	36 Rectus_L (aal); 20 Frontal_Sup_Orb_L (aal); 11 Frontal_Mid_Orb_L (aal); 10 Temporal_Pole_Sup_L (aal); 7 Frontal_Med_Orb_L (aal); 5 Frontal_Inf_Orb_L (aal); 2 Rectus_R (aal); 1 Frontal_Med_Orb_R (aal)
5	0.734	0.010	49	4.53	−57 −55 −16	32 Temporal_Inf_L (aal); 13 Temporal_Mid_L (aal); 4 Occipital_Inf_L (aal);
6	0.819	0.002	64	4.45	−6 26 56	38 Frontal_Sup_Medial_L (aal); 16 Supp_Motor_Area_L (aal); 5 Frontal_Sup_L (aal); 5 Frontal_Sup_Medial_R (aal);
7	0.821	0.014	46	4.39	39 5 56	31 Frontal_Mid_R (aal); 7 Frontal_Sup_R (aal); 5 Precentral_R (aal)
8	0.732	0.005	56	4.18	−51 −49 44	56 Parietal_Inf_L (aal)
9	0.798	0.011	48	4.02	−48 38 −1	32 Frontal_Inf_Tri_L (aal); 9 Frontal_Mid_Orb_L (aal); 7 Frontal_Inf_Orb_L (aal)

**Fig. 2:**
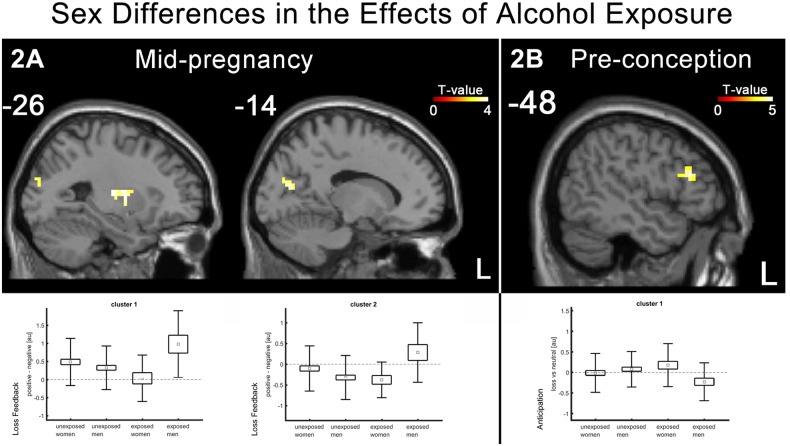
Sex differences in the effects of alcohol exposure. **A** Men exposed to alcohol in mid-pregnancy showed greater brain response to no loss (vs. loss) feedback in putamen and occipital region. **B** Women exposed to alcohol pre-conception showed greater brain response to loss (vs. no loss) anticipation in left inferior frontal cluster.

Sex differences emerged in the effects of prenatal alcohol exposure on brain function during loss feedback, where men exposed to prenatal alcohol had the greatest response to No loss feedback in the putamen (41 voxels, MNI coordinates: x = −27, y = −4, z = 8; _max_t-value = 4.17, FWEp = 0.025, ΔR^2^ = 0.122; see Fig. 2A) and occipital region (40 voxels, MNI coordinates: x = −15, y = −73, z = 17; _max_t-value = 4.05, FWEp = 0.028, ΔR^2^ = 0.126; see Fig. 2A).

When correcting the models for maternal education and maternal depression during pregnancy, there were only minor changes in the results. Exposure to alcohol during mid-pregnancy was also associated with reward feedback but not reward anticipation in the young adult offspring and individuals exposed to alcohol prenatally showed greater brain response to reward feedback than individuals not exposed to alcohol prenatally in 10 clusters (see Supplementary Fig. 6 and Supplementary Table 7), thus identifying one additional cluster when compared to the results of the simpler model. In addition, individuals exposed to alcohol prenatally showed greater brain response to No loss feedback than individuals not exposed to alcohol prenatally in 1 cluster in the right lingual gyrus (see Supplementary Fig. 7 and Supplementary Table 8). Sex differences in the effects of prenatal alcohol exposure on brain function during loss feedback, where men exposed to prenatal alcohol had the greatest response to No loss feedback not only in the putamen and occipital region, but also in left inferior temporal cluster (See Supplementary Fig. 8 and Supplementary Table 9). There were no effects of pre-conception alcohol on brain response during reward feedback or reward anticipation.

### Reward processing and cannabis use in young adulthood

**Fig. 3:**
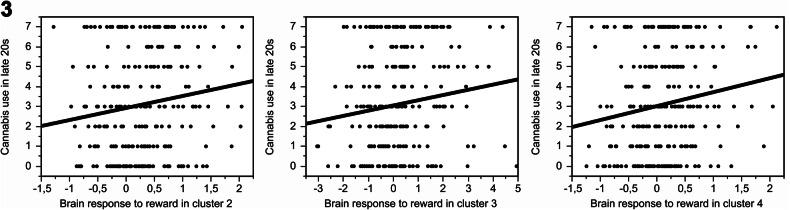
Greater brain response to reward in the three prenatal alcohol-related clusters was associated with greater cannabis use in the late 20 s. **A** Right lingual (B = 0.58, FDRp = 0.028), (**B**)—left mid-frontal (B = 0.30, FDRp = 0.050), (**C**)—left frontal superior orbital (B = 0.73, FDRp = 0.028). Since the lifetime cannabis use was reported as a category (0, 1, 2, 3–5, 6–9, 10–19, 20–39, 40 and more), we coded these from 0 to 7, where 7 reflects the greatest cannabis use.

Greater reward response in three (right lingual: beta = 0.58, FDRp = 0.028, R^2^ = 0.02, left mid-frontal: beta = 0.30, FDRp = 0.050, R^2^ = 0.02, and left frontal superior orbital: beta = 0.73, FDRp = 0.028, R^2^ = 0.03) out of the nine significant clusters was associated with greater cannabis use in the late 20 s (Fig. 3). These effects remained significant also when correcting the models for current alcohol use (right lingual: beta = 0.15, FDRp = 0.02, R^2^ = 0.10, left mid-frontal: beta = 0.14, FDRp = 0.02, R^2^ = 0.10, and left frontal superior orbital: beta = 0.16, FDRp = 0.02, R^2^ = 0.10), suggesting that the relationship between brain response to reward and cannabis use in the late 20 s is unrelated to personal alcohol use but rather to prenatally programmed remodeling of reward circuits.

### Pre-conception exposure to alcohol and reward processing in young adulthood

Exposure to alcohol pre-conception interacted with sex to predict reward anticipation but not reward feedback in the young adult offspring. Women exposed to alcohol pre-conception had the greatest response to loss vs. neutral anticipation in the left frontal inferior region (46 voxels, MNI coordinates: x = −57, y = 23, z = 23; _max_t-value = −3.15, FWEp = 0.004, ΔR^2^ = 0.061; see Fig. 2B). These results suggest that the effect of prenatal exposure to alcohol on reward feedback are pregnancy-specific.

## Discussion

We showed that even relatively moderate exposure to alcohol in mid-pregnancy but not pre-conception is associated with robust effects on brain response to reward feedback and with greater cannabis use in both men and women 30 years later. These findings are in line with those of Dodge et al. [37] who concluded that the effects of prenatal exposure to alcohol on substance use in young adulthood are not attributable to maternal drinking in general but specifically to alcohol exposure during the very sensitive period of in utero brain development. Alcohol consumed by the pregnant mother easily passes through the placenta [55] and our results support the hypothesis that even a relatively moderate exposure to alcohol can directly affect the developing brain of the fetus and have long-lasting implications for reward processing and substance use in young adulthood.

Consistently with previous research on the impact of prenatal alcohol exposure using different fMRI tasks [9–11, 13], prenatally exposed offspring had greater brain response especially in the frontal cortices. According to Fryer et al. [9], this greater brain response in prefrontal regions is suggestive of an immature pattern of prefrontal engagement or might mitigate the decreased fronto-striatal network efficiency induced by alcohol teratogenesis. Our findings of the robust and large effects of prenatal alcohol on brain response in the frontal regions is also in agreement with structural MRI studies, which linked prenatal alcohol exposure with reduced volume in the frontal lobe [12], including the middle frontal gyri in the prefrontal cortex [56].

Sex differences in the brain response to reward outcome emerged only during the no loss vs. loss contrast. Young adult men exposed to alcohol prenatally had larger brain response to no loss vs. loss in the putamen and occipital region than women exposed to prenatal alcohol. In contrast to Dhingra et al. [40], who reported higher neural sensitivity to reward in men vs. women conducting the MID task, we did not observe any sex difference among young adults without prenatal exposure to alcohol. Since alcohol affects the production and release of hormones [32, 57], the altered hormone milieu might affect the sexual differentiation of the fetus and lead to sex differences in the brain dopamine system. In fact, according to Converse et al. [58], prenatal exposure to alcohol is associated with long-term increase in dopamine receptor binding in males but not females. Since hypothyroidism was associated with small but significant increase in D2 receptor concentration in the striatum [59, 60], we speculate that the alcohol-related changes in thyroid hormones might lead to changes in dopamine receptor activity and the altered brain response to reward.

While other MID studies in individuals with substance use disorders reported altered anticipatory processing of reward [47], the current study found robust large effects of exposure to alcohol in mid-pregnancy for the hedonic aspects of reward (processing of reward outcome). Altered anticipation of reward outcomes (loss vs. neutral anticipation) was found only in the left inferior frontal region in women exposed to alcohol pre-conception. Together, these findings suggest that altered anticipatory processing of reward is unrelated to prenatal exposure to alcohol but might rather stem from other conditions related to maternal alcohol drinking preconception such as low SES, maternal anxiety or impulsivity.

Our findings also demonstrated that mid-pregnancy but not pre-conception exposure to alcohol is associated with greater cannabis use in young adulthood. Moreover, the extent of cannabis use in the late 20 s was predicted by brain response to reward feedback in three out of the nine prenatal alcohol-related clusters and these effects were independent of current alcohol use. These findings are in agreement with previous research reporting increased risk for drug addiction in the offspring exposed to alcohol prenatally [28]. In contrast to previous research [22, 23], we did not find greater alcohol drinking or cigarette smoking in the young adults exposed to alcohol prenatally. Although this is surprising, prior work has shown effects of prenatal alcohol exposure on endocannabinoid receptors [28], which may explain the link between moderate alcohol drinking during pregnancy and altered cannabis use in offspring. It is also important to note that the questionnaire assessed lifetime cannabis use but only probed alcohol and cigarette use in the past 30 days. Therefore, the presence of significant effect on lifetime cannabis use but not recent alcohol and cigarette use might not be related to the type of substance but to the long-term vs. relatively brief character of the substance use measures and the fact that the long-term effects of prenatal alcohol exposure would likely manifest in the lifetime pattern of behavior rather than a specific period, which might be much more influenced by recent stressful life events than prenatal factors.

Our findings are limited by the relatively small sample size of the exposed groups (preconception exposure n = 60 vs. n = 138 non-exposed; mid-pregnancy exposure n = 33 vs. n = 161 non-exposed). However, a lower prevalence of alcohol exposure vs. non-exposure is expected in a prenatal birth cohort study. Thus, future research should replicate our exploratory findings in an independent sample with a possibly larger sample size. Another limitation is the fact that the questions regarding substance use in young adulthood we used and described in the Methods are not part of a standardized questionnaire. Further, given the fact that the longitudinal data from a prenatal birth cohort allow us to describe only associations between the early life factors and outcomes in adulthood, there might be also alternative explanations of the relationship between maternal alcohol drinking during mid-pregnancy and reward processing and cannabis use in young adulthood. For example, the mothers drinking in mid-pregnancy might also have different parenting styles, which can in turn increase the propensity to higher cannabis use in offspring. Alternatively, mothers and offspring may have shared genetic risk factors that independently shape drinking behavior during pregnancy in the mothers and offspring reward-related brain function as well as offspring substance use patterns. Given that our cannabis use assessment probed lifetime use, the current study does not allow us to make any conclusions regarding the specific timing of when the effects of prenatal alcohol on cannabis use may emerge. Future research should include more detailed longitudinal data to assess this.

Still, the design of our study is strong. The information regarding alcohol exposure was collected prospectively in the early 90 s and thus our findings are not affected by any retrospective bias such as in other studies [20], all mothers reported their current alcohol use in mid-pregnancy and thus our findings are not confounded by a lack of time-specificity, and we compared the impact of mid-pregnancy vs. preconception alcohol exposure on reward processing and substance use in young adulthood, allowing to disentangle the pregnancy-specific effects of alcohol exposure from other possibly confounding factors associated with alcohol drinking in general. Moreover, given the definition of our prenatal birth cohort, all participants had a very similar age (born 1991 or 1992), were of European ancestry, growing-up in the same area, and due to the early post-communist era in Czechoslovakia in the early 90 s, they were born into families with a very similar socioeconomic status. The number of men and women was relatively even in each group, allowing to assess the interaction between prenatal alcohol exposure and sex. While the self-reported nature of our prenatal alcohol consumption measure might be seen as a limitation [61], given the stigma associated with maternal drinking during pregnancy, the mothers might be more sincere while anonymously completing a self-report questionnaire rather than in any interview-based measures. Based on Howlett et al. [62], who compared the prevalence of alcohol consumption during pregnancy using self-report versus blood-based biomarker (carbohydrate deficient transferrin [CDT], a marker of alcohol exposure, which normalizes 2–3 weeks from abstinence), the estimates for self-report and CDT were not significantly different.

Overall, our findings, based on 30 years’ worth of data on a prenatal birth cohort, suggest that even relatively moderate exposure to alcohol during pregnancy, a critical sensitive period for brain development, might alter neural reward processing in the offspring and contribute to the intergenerational transmission of risk for substance use disorders. This is the first prospective longitudinal study testing the impact of prenatal and pre-conception exposure to alcohol on brain response to reward processing and substance use in young adulthood. While a number of studies reported altered neurodevelopment and behavior in FASD, this is the first study demonstrating that even moderate levels of alcohol drinking during pregnancy have long-lasting effects on brain function and risk of cannabis use in the offspring. Therefore, our study has critical implications for public health messaging on possible harms related to drinking during pregnancy.

### Supplementary information

Supplementary Material

## Data Availability

The MATLAB code used for fMRI data preprocessing and analyses is available in the Supplementary Methods.

## Data availability

The data that support the findings of this study are available from the corresponding author (KM) upon reasonable request.
