# Supplementary material for: Prenatal exposure to alcohol and its impact on reward processing and substance use in adulthood
Source: Transl Psychiatry. 2024 May 28;14:220. doi: 10.1038/s41398-024-02941-9 (PMC11133468; doi:10.1038/s41398-024-02941-9)

**SUPPLEMENTARY MATERIALS**

**Supplementary Table 1:**  The schema of adjustment of the time limit for probe. The minimal and maximal duration of probe were set to 150 ms and 500 ms, resp. The initial value was set according to results of pre-MR training session.

| actual ratio of correct answers [%] | 0-5 | 5-15 | 15-25 | 25-35 | 35-45 | 45-55 | 55-65 | 65-75 | 75-85 | 85-95 | 95-100 |
| --- | --- | --- | --- | --- | --- | --- | --- | --- | --- | --- | --- |
| probe limit time adjustment [ms] | +70 | +60 | +50 | +40 | +30 | +20 | 0 | -20 | -30 | -40 | -50 |

**Supplementary Figure 1:** Example of the stimuli creating one trial during the Reward feedback condition of the MID fMRI task.

| 2s  CUE | 1s to 4s  ANTICIPATION | up to current time limit  PROBE | 2s – PROBE time  FEEDBACK |
| --- | --- | --- | --- |
| cue text in probe shape | fixation cross | probe image | feedback text |
| 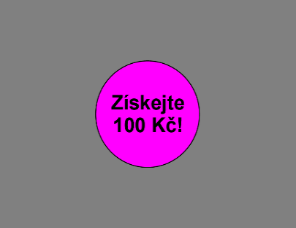 | 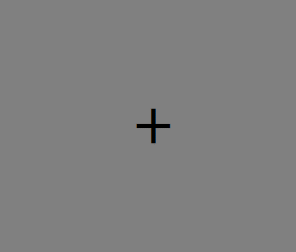 | 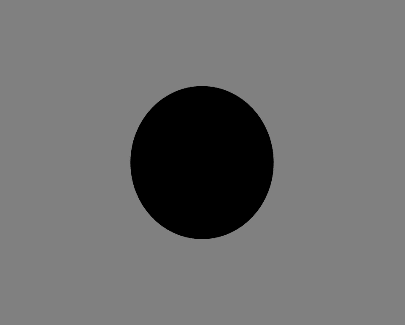 | 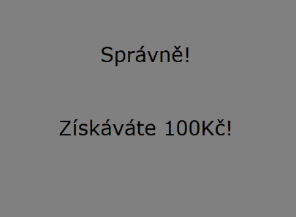 |

**Supplementary Table 2:** Stimuli details. We used texts in Czech; English translation is available in brackets.

| **Trial type** | **Cue text** | **Probe image** | **Feedback text** | |
| --- | --- | --- | --- | --- |
|  |  |  | **correct response** | **incorrect response** |
| **REWARD** | Získejte 100 Kč!  (Win 100 CZK!) |  | Správně!  Získáváte 100 Kč  (Correct response!  You earn 100 CZK!) | Zmáčkli jste tlačítko moc brzy! / Zmáčkli jste tlačítko moc pomalu!  Nezískáváte 100 Kč!  (You pressed too soon! / You pressed too slow!  You did not earn 100 CZK!) |
| **LOSS** | Neztraťte 100 Kč!  (Don’t lose 100 CZK!) |  | Správně!  Neztrácíte 100 Kč!  (Correct response!  You keep 100 CZK!) | Zmáčkli jste tlačítko moc brzy! / Zmáčkli jste tlačítko moc pomalu!  Ztrácíte 100 Kč!  (You pressed too soon! / You pressed too slow!  You lose 100 CZK!) |
| **NEUTRAL** | V sázce nejsou žádné peníze.  (No money at stake.) |  | Správně!  Teď nejsou v sázce žádné peníze.  (Correct response!  No money at stake.) | Zmáčkli jste tlačítko moc brzy! / Zmáčkli jste tlačítko moc pomalu!  Teď nejsou v sázce žádné peníze.  (You pressed too soon! / You pressed too slow!  No money at stake.) |

**Supplementary Figure 2:** Brain response to the contrast Anticipation of reward vs. Anticipation of neutral, which survived the initial voxel-wise uncorrected threshold of p<0.001 as well as the cluster-level threshold of FWEp<0.05.


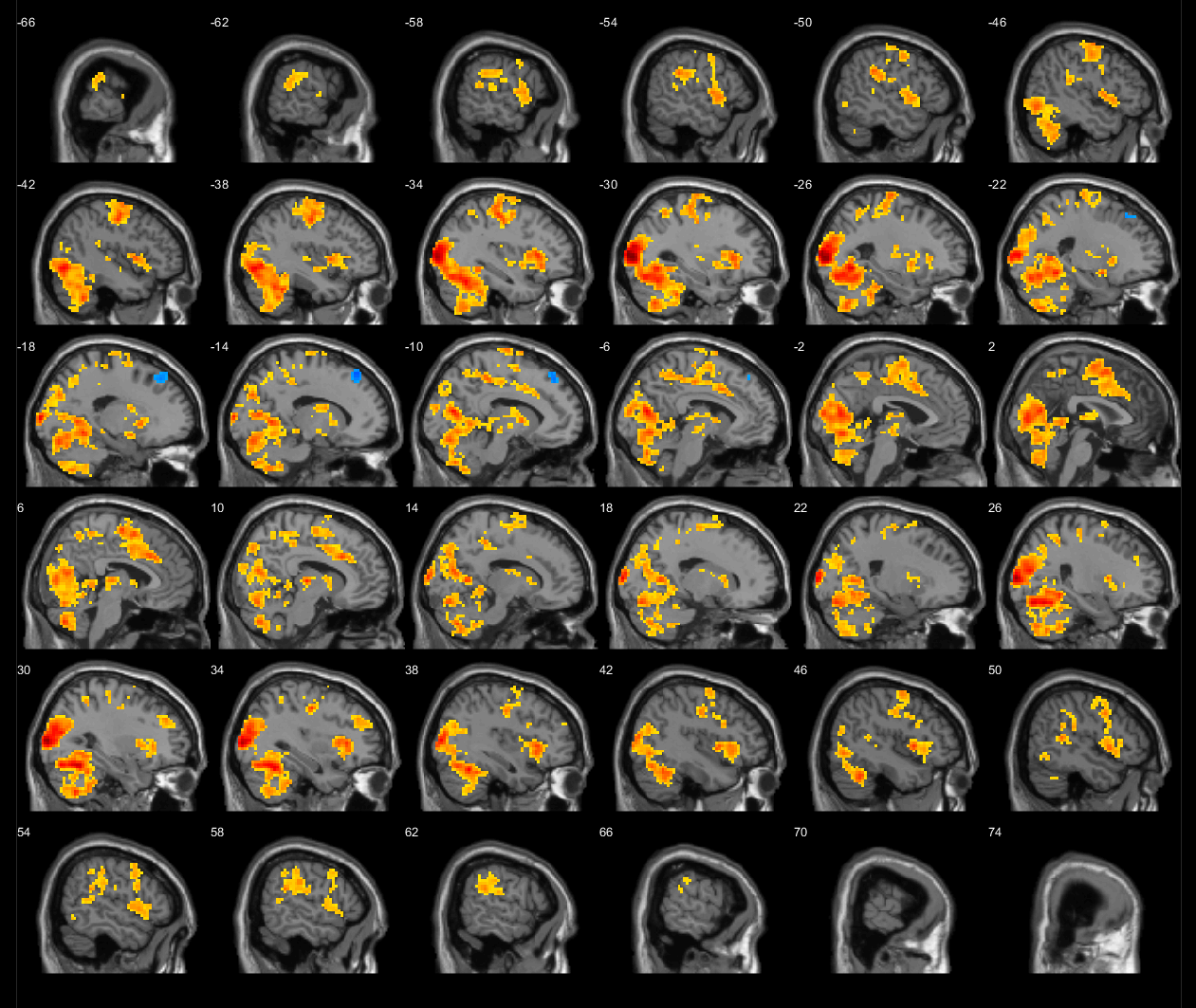


**Supplementary Table 3:** Brain response to the contrast Anticipation of reward vs. Anticipation of neutral, which survived the initial voxel-wise uncorrected threshold of p<0.001 as well as the cluster-level threshold of FWEp<0.05.

| # cluster | cluster – level | | | | Number of voxels per AAL region |
| --- | --- | --- | --- | --- | --- |
|  | p FWE | # voxels | T-value in maximum | x,y,z (mm) |  |
| 1 | <0.001 | 9686 | 9.45 | -30 -91 5 | 477 Occipital_Mid_L; 414 Precentral_L; 386 Cerebelum_6_L; 362 Occipital_Mid_R; 360 Lingual_L; 344 Cerebelum_Crus1_L; 339 Cerebelum_6_R; 338 Fusiform_L; 308 Cerebelum_8_R; 301 Cerebelum_8_L; 296 Calcarine_L; 260 Lingual_R; 239 Cuneus_R; 232 Fusiform_R; 231 Calcarine_R; 220 Cerebelum_Crus1_R; 217 Supp_Motor_Area_L; 205 Cingulum_Mid_R; 203 Supp_Motor_Area_R; 192 Occipital_Sup_R; 174 Cingulum_Mid_L; 169 Insula_L; 167 Cuneus_L; 161 Postcentral_L; 150 Occipital_Sup_L; 149 Occipital_Inf_L; 120 Cerebelum_Crus2_L; 106 Cerebelum_4_5_L; 105 Cerebelum_7b_L; 98 Precuneus_R; 90 Occipital_Inf_R; 90 Precuneus_L; 87 Putamen_L; 82 Parietal_Sup_L; 69 Cerebelum_4_5_R; 64 Rolandic_Oper_L; 62 Cerebelum_Crus2_R; 57 Frontal_Inf_Oper_L; 55 Thalamus_R; 54 Frontal_Sup_R; 54 Paracentral_Lobule_L; 54 Thalamus_L; 51 Temporal_Inf_R; 45 Cerebelum_9_L; 43 Caudate_L; 43 Cerebelum_7b_R; 41 Cerebelum_9_R; 40 Frontal_Sup_L; 40 Temporal_Inf_L; 32 Frontal_Inf_Orb_L; 32 Frontal_Inf_Tri_L; 28 Paracentral_Lobule_R; 28 Temporal_Pole_Sup_L; 28 Temporal_Sup_L; 24 Temporal_Mid_R; 23 Parietal_Sup_R; 21 Precentral_R; 19 Temporal_Mid_L; 14 Parietal_Inf_L; 14 Parietal_Inf_R; 13 Postcentral_R; 11 ParaHippocampal_L; 10 Amygdala_L; 10 Cingulum_Ant_R; 8 Pallidum_L; 5 Hippocampus_L; 5 Olfactory_L; 4 Cingulum_Post_R; 4 ParaHippocampal_R; 3 Cerebelum_10_L; 3 Cerebelum_3_R; 3 Frontal_Sup_Medial_L; 3 Frontal_Sup_Medial_R; 3 Heschl_L; 2 Angular_R; 2 Cerebelum_10_R; 2 Frontal_Mid_L; 1 Cingulum_Ant_L; 1 SupraMarginal_L |
| 2 | <0.001 | 534 | 6.28 | 36 20 5 | 172 Insula_R; 53 Frontal_Inf_Orb_R; 52 Frontal_Inf_Oper_R; 51 Rolandic_Oper_R; 47 Putamen_R; 39 Frontal_Inf_Tri_R; 32 Caudate_R; 15 Temporal_Pole_Sup_R; 3 Thalamus_R; 2 Temporal_Sup_R; 1 Heschl_R; 1 Pallidum_R |
| 3 | <0.001 | 323 | 6.20 | 33 -13 44 | 195 Precentral_R; 44 Frontal_Mid_R; 33 Frontal_Inf_Oper_R; 7 Postcentral_R; 5 Frontal_Inf_Tri_R; 4 Frontal_Sup_R; |
| 4 | <0.001 | 258 | 5.51 | -51 -37 32 | 130 SupraMarginal_L; 68 Temporal_Sup_L; 36 Postcentral_L; 8 Parietal_Inf_L; 3 Rolandic_Oper_L; 1 Temporal_Mid_L |
| 5 | <0.001 | 334 | 5.20 | 51 -43 11 | 243 SupraMarginal_R; 59 Temporal_Sup_R; 21 Temporal_Mid_R; 4 Postcentral_R; 4 Rolandic_Oper_R; 1 Parietal_Inf_R; |
| 6 | <0.001 | 81 | 4.80 | 30 41 32 | 81 Frontal_Mid_R |
| 7 | <0.001 | 60 | 4.78 | -27 32 35 | 56 Frontal_Mid_L; 4 Frontal_Sup_L |
| 8 | 0.002 | 53 | -3.13 | -51 -61 38 | 31 Angular_L; 4 Parietal_Sup_L |
| 9 | <0.001 | 69 | -3.12 | -18 32 47 | 49 Frontal_Sup_L; 11 Frontal_Sup_Medial_L; 2 Frontal_Mid_L |

**Supplementary Figure 3:** Brain response to the contrast Anticipation of loss vs. Anticipation of neutral, which survived the initial voxel-wise uncorrected threshold of p<0.001 as well as the cluster-level threshold of FWEp<0.05.


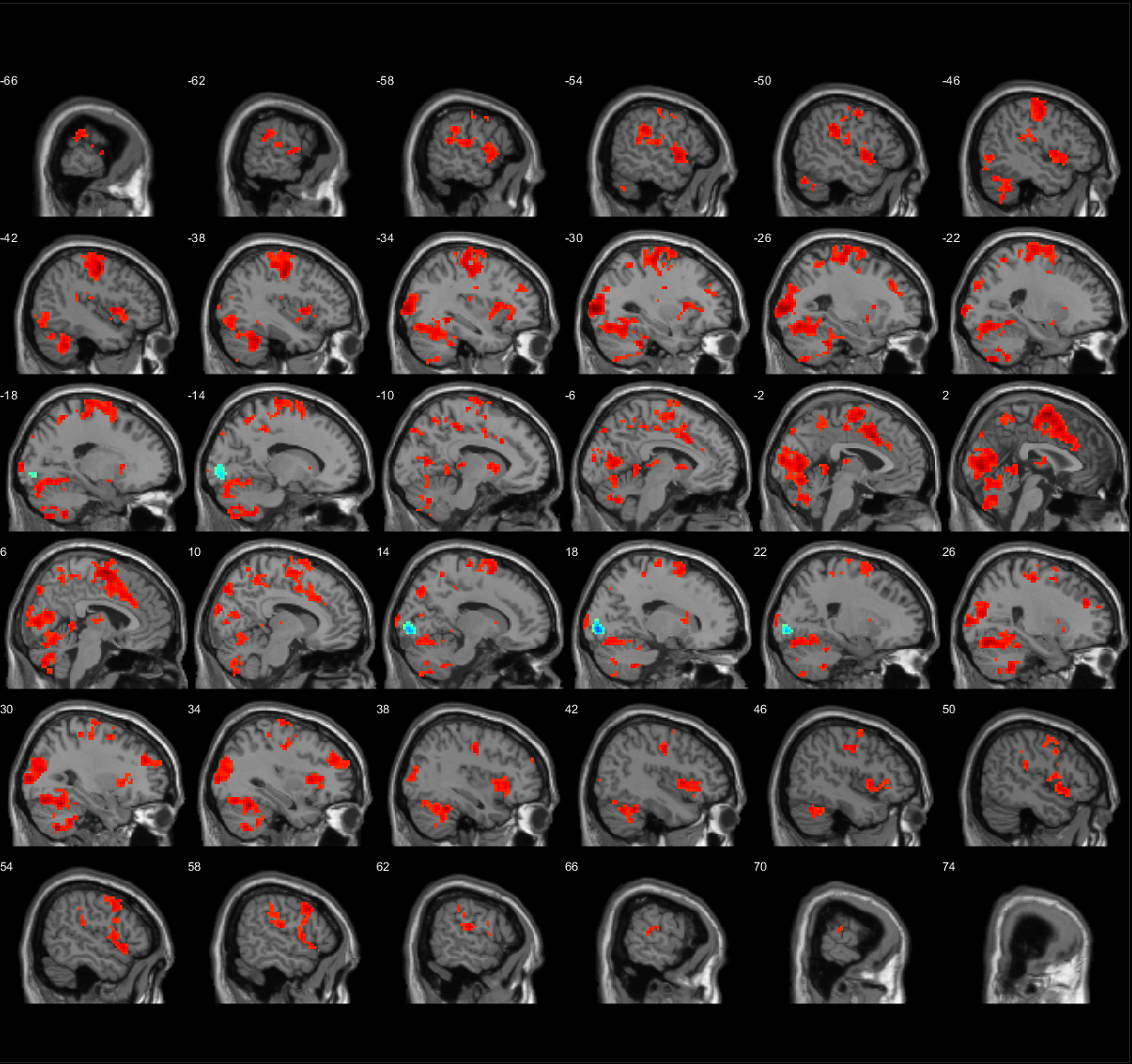


**Supplementary Table 4:** Brain response to the contrast Anticipation of loss vs. Anticipation of neutral, which survived the initial voxel-wise uncorrected threshold of p<0.001 as well as the cluster-level threshold of FWEp<0.05.

| # cluster | cluster – level | | | | Number of voxels per AAL region |
| --- | --- | --- | --- | --- | --- |
|  | p FWE | # voxels | T-value in maximum | x,y,z (mm) |  |
| 1 | <0.001 | 1650 | 6.71 | -42 -10 53 | 342 Precentral_L; 246 Supp_Motor_Area_R; 225 Postcentral_L; 188 Supp_Motor_Area_L; 127 Frontal_Sup_L; 123 Cingulum_Mid_R; 93 Cingulum_Mid_L; 63 Frontal_Sup_R; 54 Paracentral_Lobule_L;19 Cingulum_Ant_L; 15 Precentral_R; 12 Cingulum_Ant_R; 10 Frontal_Mid_R; 6 Frontal_Sup_Medial_L; 4 Frontal_Mid_L; 4 Parietal_Inf_L; 3 Frontal_Sup_Medial_R; 2 SupraMarginal_L; 1 Parietal_Sup_L |
| 2 | <0.001 | 357 | 6.53 | -30 -94 5 | 218 Occipital_Mid_L; 40 Occipital_Sup_L; 32 Cuneus_R; 22 Precuneus_R; 15 Precuneus_L; 3 Occipital_Inf_L; 3 Parietal_Sup_R |
| 3 | <0.001 | 2498 | 5.98 | 0 -85 8 | 227 Cerebelum_6_L; 223 Cerebelum_6_R; 200 Cerebelum_Crus1_L; 172 Occipital_Mid_R; 169 Calcarine_L; 159 Fusiform_L; 131 Cerebelum_8_L; 130 Cerebelum_8_R; 117 Fusiform_R; 103 Cerebelum_Crus1_R; 102 Lingual_L; 92 Calcarine_R; 63 Cuneus_L; 55 Lingual_R; 49 Cuneus_R; 45 Occipital_Inf_L; 42 Cerebelum_Crus2_L; 38 Occipital_Sup_R; 29 Cerebelum_7b_L; 23 Cerebelum_4_5_R; 23 Cerebelum_9_L; 17 Cerebelum_7b_R; 17 Cerebelum_9_R; 15 Cerebelum_Crus2_R; 14 Cerebelum_4_5_L; 9 Occipital_Mid_L; 5 ParaHippocampal_L; 5 Temporal_Inf_L; 1 Cerebelum_10_R; 1 Occipital_Inf_R; 1 Precuneus_R |
| 4 | <0.001 | 322 | 5.46 | -54 5 -1 | 100 Insula_L; 52 Rolandic_Oper_L; 35 Temporal_Sup_L; 31 Frontal_Inf_Oper_L; 31 Temporal_Pole_Sup_L; 16 Frontal_Inf_Orb_L; 14 Frontal_Inf_Tri_L; 9 Putamen_L; 2 Amygdala_L; 1 Postcentral_L |
| 5 | <0.001 | 247 | 5.39 | -51 -34 32 | 103 SupraMarginal_L; 68 Temporal_Sup_L; 25 Postcentral_L; 16 Rolandic_Oper_L; 9 Insula_L; 5 Parietal_Inf_L |
| 6 | <0.001 | 112 | 5.35 | 60 -19 17 | 79 SupraMarginal_R; 17 Temporal_Sup_R; 8 Postcentral_R; 8 Rolandic_Oper_R |
| 7 | <0.001 | 499 | 5.18 | 33 17 5 | 140 Insula_R; 125 Precentral_R; 47 Rolandic_Oper_R; 34 Frontal_Inf_Oper_R; 26 Temporal_Pole_Sup_R; 22 Frontal_Inf_Orb_R; 16 Frontal_Inf_Tri_R; 16 Frontal_Mid_R; 13 Putamen_R; 8 Postcentral_R; 7 Caudate_R |
| 8 | <0.001 | 69 | 4.93 | 27 -19 56 | 31 Precentral_R; 24 Postcentral_R; 2 Frontal_Sup_R |
| 9 | 0.003 | 49 | 4.90 | -9 -28 5 | 23 Thalamus_L; 18 Thalamus_R |
| 10 | <0.001 | 67 | 4.72 | 3 -43 -1 | 9 Cerebelum_4_5_L; 7 Lingual_L; 7 Lingual_R; 3 Calcarine_L; 3 Cingulum_Post_R; 1 Precuneus_R |
| 11 | <0.001 | 101 | 4.70 | 27 41 29 | 97 Frontal_Mid_R |
| 12 | <0.001 | 168 | 4.61 | -9 -34 50 | 65 Precuneus_R; 35 Precuneus_L; 25 Cingulum_Mid_L; 11 Paracentral_Lobule_R; 10 Paracentral_Lobule_L; 7 Cingulum_Mid_R; 4 Parietal_Sup_R |
| 13 | <0.001 | 61 | 4.39 | -21 -55 62 | 34 Parietal_Sup_L; 19 Precuneus_L; 6 Parietal_Inf_L |
| 14 | 0.011 | 40 | 4.20 | -9 5 5 | 25 Caudate_L; 5 Putamen_L; 2 Pallidum_L |
| 15 | 0.004 | 48 | 4.10 | -27 32 35 | 43 Frontal_Mid_L; 5 Frontal_Sup_L |
| 16 | 0.006 | 44 | -3.15 | -12 -91 -10 | 17 Lingual_L; 8 Calcarine_L; 8 Occipital_Mid_L; 6 Occipital_Sup_L; 1 Fusiform_L |
| 17 | 0.001 | 59 | -3.13 | 21 -91 2 | 28 Lingual_R; 26 Calcarine_R; 2 Cuneus_R; 2 Occipital_Inf_R |

**Supplementary Figure 4:** Brain response to the contrast Reward feedback vs. No reward feedback, which survived the initial voxel-wise uncorrected threshold of p<0.001 as well as the cluster-level threshold of FWEp<0.05.


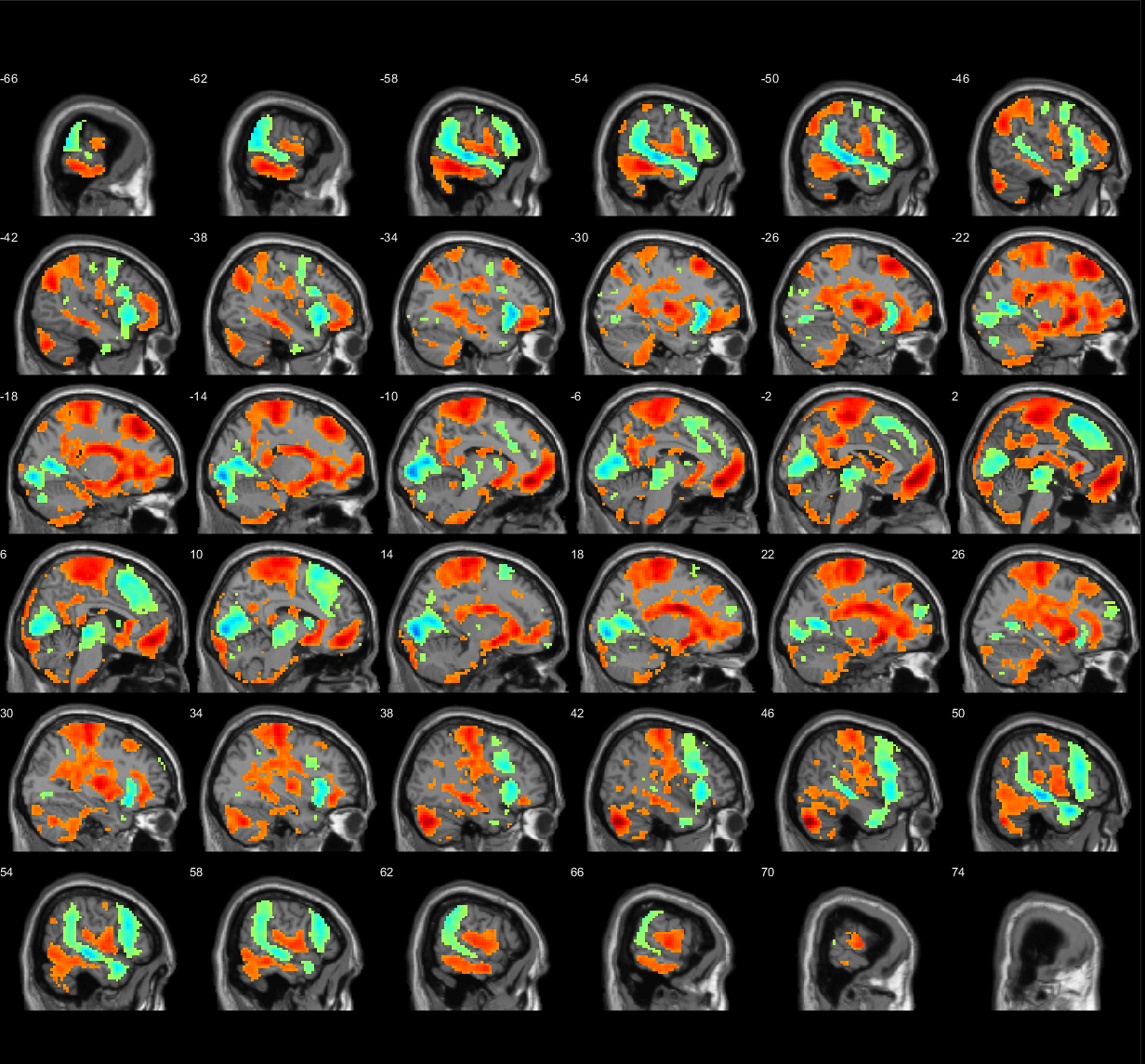


**Supplementary Table 5:** Brain response to the contrast Reward feedback vs. No reward feedback, which survived the initial voxel-wise uncorrected threshold of p<0.001 as well as the cluster-level threshold of FWEp<0.05.

| # cluster | cluster – level | | | | Number of voxels per AAL region |
| --- | --- | --- | --- | --- | --- |
|  | p FWE | # voxels | T-value in maximum | x,y,z (mm) |  |
| 1 | <0.001 | 27320 | 13.09 | -6 44 -10 | 739 Temporal_Mid_L; 662 Postcentral_R; 561 Precentral_R; 538 Temporal_Inf_R; 480 Temporal_Mid_R; 478 Temporal_Sup_R; 471 Frontal_Sup_L; 451 Precuneus_L; 432 Frontal_Inf_Tri_L; 418 Postcentral_L; 408 Frontal_Mid_L; 394 Supp_Motor_Area_R; 393 Calcarine_L; 381 Cerebelum_Crus1_R; 371 Paracentral_Lobule_L; 367 Calcarine_R; 344 Precentral_L; 344 Temporal_Inf_L; 343 Frontal_Sup_Medial_L; 307 Parietal_Inf_L; 306 Frontal_Sup_Medial_R; 304 Frontal_Inf_Tri_R; 291 Frontal_Inf_Orb_L; 282 Lingual_R; 274 Lingual_L; 273 Frontal_Mid_R; 270 Temporal_Sup_L; 267 Frontal_Sup_R; 267 Putamen_L; 263 Frontal_Inf_Oper_R; 256 Putamen_R; 249 Insula_R; 244 Angular_L; 239 Cerebelum_8_L; 236 SupraMarginal_R; 230 Cerebelum_Crus2_R; 224 Rolandic_Oper_R; 222 Parietal_Sup_L; 216 Paracentral_Lobule_R; 216 Supp_Motor_Area_L; 215 Cingulum_Mid_R; 214 Precuneus_R; 212 Cerebelum_Crus1_L; 206 Caudate_R; 201 Insula_L; 199 Frontal_Inf_Orb_R; 194 Parietal_Sup_R; 187 Frontal_Med_Orb_L; 180 Frontal_Mid_Orb_L; 179 Caudate_L; 179 Frontal_Med_Orb_R; 170 Hippocampus_L; 152 Cingulum_Mid_L; 152 Cuneus_L; 147 Cerebelum_8_R; 139 SupraMarginal_L; 136 Rectus_L; 134 Rolandic_Oper_L; 133 Cerebelum_Crus2_L; 132 Frontal_Sup_Orb_L; 127 Hippocampus_R; 121 Cuneus_R; 115 Frontal_Inf_Oper_L; 114 Fusiform_R; 110 Fusiform_L; 110 Rectus_R; 110 Temporal_Pole_Sup_L; 106 ParaHippocampal_R; 102 Temporal_Pole_Mid_R; 100 Cerebelum_4_5_L; 94 Thalamus_R; 91 ParaHippocampal_L; 86 Cingulum_Ant_L; 84 Angular_R; 82 Cingulum_Post_L; 81 Temporal_Pole_Sup_R; 78 Occipital_Mid_L; 67 Occipital_Inf_L; 62 Cerebelum_9_L; 61 Cerebelum_9_R; 58 Cingulum_Ant_R; 57 Thalamus_L; 55 Olfactory_R; 51 Parietal_Inf_R; 49 Olfactory_L; 47 Frontal_Sup_Orb_R; 44 Frontal_Mid_Orb_R; 44 Heschl_R; 40 Amygdala_L; 39 Cerebelum_6_L; 36 Cingulum_Post_R; 35 Occipital_Inf_R; 35 Temporal_Pole_Mid_L; 33 Cerebelum_4_5_R; 31 Cerebelum_6_R; 30 Occipital_Sup_L; 25 Amygdala_R; 24 Cerebelum_10_R; 22 Cerebelum_7b_L; 21 Cerebelum_7b_R; 21 Heschl_L; 17 Cerebelum_10_L; 11 Pallidum_R; 9 Occipital_Sup_R; 9 Pallidum_L; 6 Occipital_Mid_R; 3 Cerebelum_3_R; 2 Cerebelum_3_L |
| 2 | <0.001 | 79 | -3.15 | -51 -22 59 | 62 Postcentral_L; 3 Precentral_L |
| 3 | <0.001 | 92 | -3.13 | 21 50 29 | 44 Frontal_Sup_R; 38 Frontal_Mid_R |

**Supplementary Figure 5:** Brain response to the contrast No loss feedback vs. Loss feedback, which survived the initial voxel-wise uncorrected threshold of p<0.001 as well as the cluster-level threshold of FWEp<0.05.


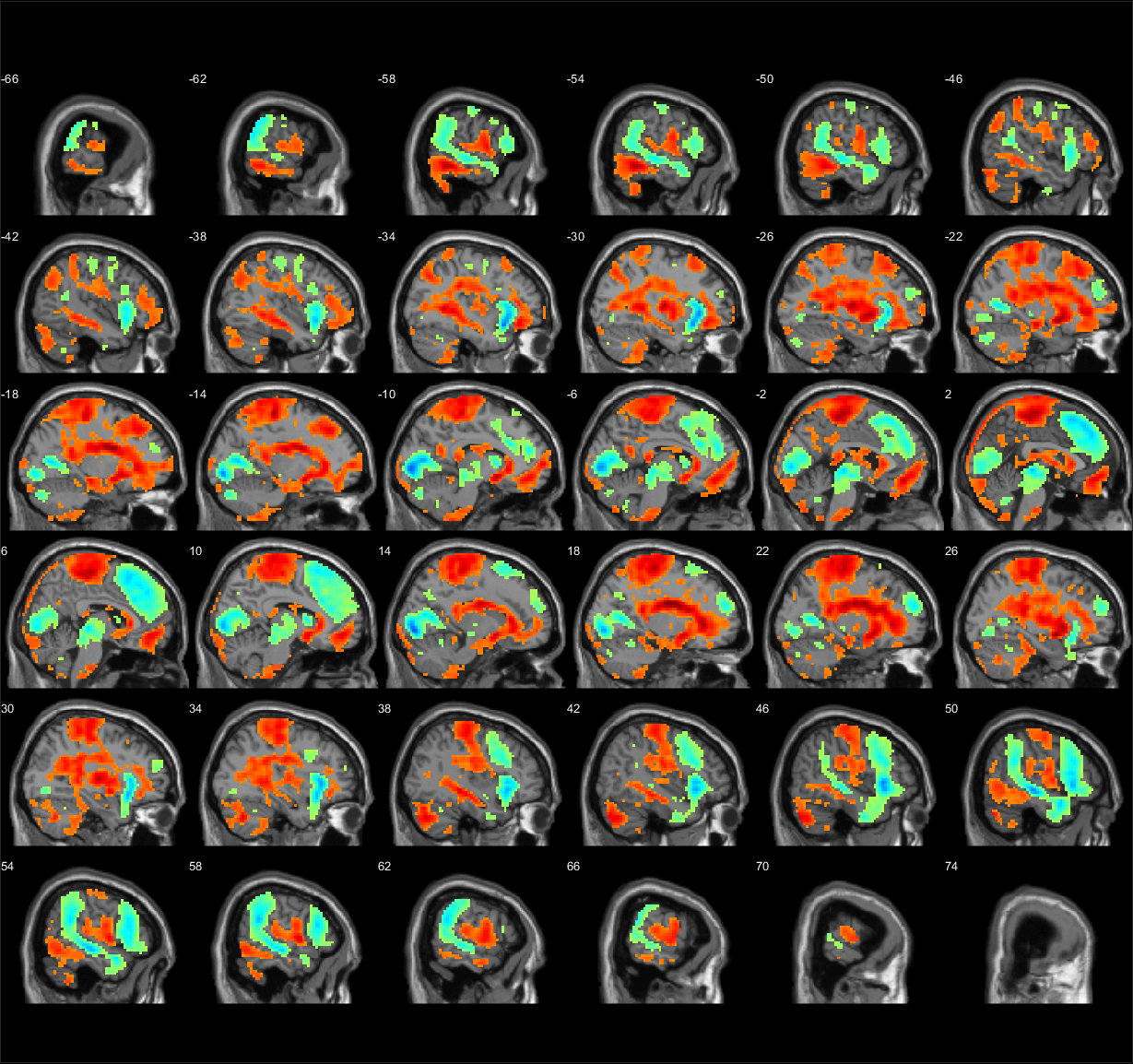


**Supplementary Table 6:** Brain response to the contrast No loss feedback vs. Loss feedback, which survived the initial voxel-wise uncorrected threshold of p<0.001 as well as the cluster-level threshold of FWEp<0.05.

| # cluster | cluster – level | | | | Number of voxels per AAL region |
| --- | --- | --- | --- | --- | --- |
|  | p FWE | # voxels | T-value in maximum | x,y,z (mm) |  |
| 1 | <0.001 | 26463 | 12.33 | 18 -10 26 | 664 Postcentral_R; 613 Precentral_R; 600 Temporal_Mid_L; 499 Temporal_Mid_R; 456 Temporal_Sup_R; 454 Frontal_Sup_Medial_L; 437 Frontal_Sup_Medial_R; 406 Frontal_Inf_Tri_L; 405 Supp_Motor_Area_R; 396 Calcarine_L; 390 Postcentral_L; 369 Calcarine_R; 369 Frontal_Mid_R; 368 Precuneus_L; 367 Paracentral_Lobule_L; 367 Temporal_Inf_R; 361 Frontal_Sup_L; 342 Frontal_Inf_Tri_R; 338 Temporal_Inf_L; 330 SupraMarginal_R; 304 Frontal_Mid_L; 300 Frontal_Inf_Oper_R; 283 Frontal_Sup_R; 277 Parietal_Sup_L; 266 Putamen_L; 260 Frontal_Inf_Orb_R; 256 Cerebelum_Crus1_R; 249 Putamen_R; 248 Lingual_R; 247 Frontal_Inf_Orb_L; 240 Insula_R; 234 Lingual_L; 231 Cerebelum_Crus2_R; 226 Insula_L; 224 Paracentral_Lobule_R; 219 Temporal_Sup_L; 217 Rolandic_Oper_R; 213 Supp_Motor_Area_L; 204 Parietal_Inf_L; 197 Caudate_R; 197 Cerebelum_8_L; 195 Angular_L; 177 Cingulum_Ant_R; 173 Caudate_L; 171 Hippocampus_L; 168 Parietal_Sup_R; 166 Cerebelum_Crus1_L; 155 Frontal_Mid_Orb_L; 149 Precentral_L; 147 Frontal_Med_Orb_L; 145 Temporal_Pole_Sup_R; 144 SupraMarginal_L; 136 Frontal_Med_Orb_R; 135 Cingulum_Ant_L; 132 Occipital_Mid_L; 130 Precuneus_R; 128 Hippocampus_R; 125 Cingulum_Mid_R; 124 Parietal_Inf_R; 117 Temporal_Pole_Mid_R; 115 Fusiform_R; 111 Cerebelum_8_R; 108 Rolandic_Oper_L; 105 Frontal_Sup_Orb_L; 103 Rectus_L; 102 Temporal_Pole_Sup_L; 98 Cuneus_L; 96 Thalamus_R; 86 Angular_R; 81 Rectus_R; 78 Frontal_Inf_Oper_L; 76 Thalamus_L; 75 ParaHippocampal_R; 71 Cerebelum_Crus2_L; 67 ParaHippocampal_L; 63 Fusiform_L; 60 Cuneus_R; 59 Cerebelum_9_L; 53 Cerebelum_4_5_R; 49 Cingulum_Mid_L; 47 Occipital_Inf_L; 46 Cerebelum_9_R; 46 Olfactory_L; 40 Olfactory_R; 35 Frontal_Sup_Orb_R; 35 Heschl_R; 33 Cerebelum_4_5_L; 33 Occipital_Sup_L; 30 Cerebelum_6_R; 27 Amygdala_L; 24 Occipital_Inf_R; 23 Temporal_Pole_Mid_L; 20 Amygdala_R; 20 Cerebelum_6_L; 20 Frontal_Mid_Orb_R; 19 Cerebelum_7b_L; 18 Cerebelum_7b_R; 16 Cerebelum_10_R; 16 Pallidum_L; 15 Pallidum_R; 14 Cerebelum_10_L; 12 Cerebelum_3_R; 11 Cingulum_Post_L; 11 Cingulum_Post_R; 11 Heschl_L; 3 Occipital_Mid_R; 3 Occipital_Sup_R; 1 Cerebelum_3_L |
| 2 | 0.008 | 48 | 4.71 | -3 -34 35 | 25 Cingulum_Mid_L; 17 Cingulum_Post_L; 6 Cingulum_Mid_R |
| 3 | <0.001 | 131 | -3.17 | -24 -73 -25 | 74 Cerebelum_Crus1_L; 55 Cerebelum_Crus2_L |
| 4 | 0.009 | 47 | -3.17 | 30 -52 -28 | 28 Cerebelum_6_R; 14 Cerebelum_4_5_R |
| 5 | 0.018 | 41 | -3.14 | 6 -58 -37 | 10 Cerebelum_9_L; 2 Cerebelum_8_L |
| 6 | <0.001 | 98 | -3.14 | -30 -1 53 | 74 Precentral_L; 7 Frontal_Mid_L |
| 7 | <0.001 | 150 | -3.13 | -60 -19 41 | 85 Postcentral_L; 35 Precentral_L; 4 Parietal_Inf_L; |
| 8 | <0.001 | 110 | -3.12 | -27 62 23 | 66 Frontal_Mid_L; 43 Frontal_Sup_L |

**Supplementary Table 7** – Description of the 10 significant clusters showing greater brain response to reward in the young adults prenatally exposed to alcohol (independently of sex, maternal education and maternal depression during pregnancy).

| # cluster |  | cluster – level | | | | Number of voxels per AAL region |
| --- | --- | --- | --- | --- | --- | --- |
|  | Hedges’ g | p FWE | # voxels | T-value in maximum | x,y,z {mm} |  |
| 1 | 0.990 | <0.001 | 82 | 5.36 | -42 20 26 | 77 Frontal_Inf_Tri_L; 1 Frontal_Inf_Oper_L |
| 2 | 0.908 | 0.004 | 52 | 4.84 | 15 -94 -10 | 43 Lingual_R; 5 Calcarine_R |
| 3 | 0.843 | <0.001 | 122 | 4.63 | -48 8 41 | 66 Frontal_Mid_L; 52 Precentral_L |
| 4 | 0.819 | <0.001 | 107 | 4.41 | -3 47 -19 | 29 Rectus_L; 28 Frontal_Sup_Orb_L; 12 Frontal_Mid_Orb_L; 12 Temporal_Pole_Sup_L; 8 Frontal_Med_Orb_L; 7 Frontal_Inf_Orb_L |
| 5 | 0.364 | 0.025 | 37 | 4.30 | 42 5 62 | 26 Frontal_Mid_R; 5 Frontal_Sup_R; 4 Precentral_R |
| 6 | 0.588 | 0.002 | 61 | 4.29 | 3 -22 56 | 23 Paracentral_Lobule_R; 15 Paracentral_Lobule_L; 10 Supp_Motor_Area_R |
| 7 | 0.774 | 0.017 | 40 | 4.27 | -18 53 14 | 35 Frontal_Sup_L; 3 Frontal_Sup_Orb_L; 2 Frontal_Mid_L |
| 8 | 0.813 | <0.001 | 83 | 4.24 | -30 47 5 | 41 Frontal_Inf_Tri_L; 26 Frontal_Mid_L; 8 Frontal_Mid_Orb_L; 7 Frontal_Inf_Orb_L; |
| 9 | 0.750 | 0.013 | 42 | 4.23 | -51 -49 44 | 42 Parietal_Inf_L |
| 10 | 0.798 | 0.006 | 49 | 4.08 | -6 26 56 | 31 Frontal_Sup_Medial_L; 13 Supp_Motor_Area_L; 5 Frontal_Sup_L |

**Supplementary Figure 6 -** Ten significant clusters showing greater brain response to reward in the young adults prenatally exposed to alcohol (independently of sex, maternal education and maternal depression during pregnancy).


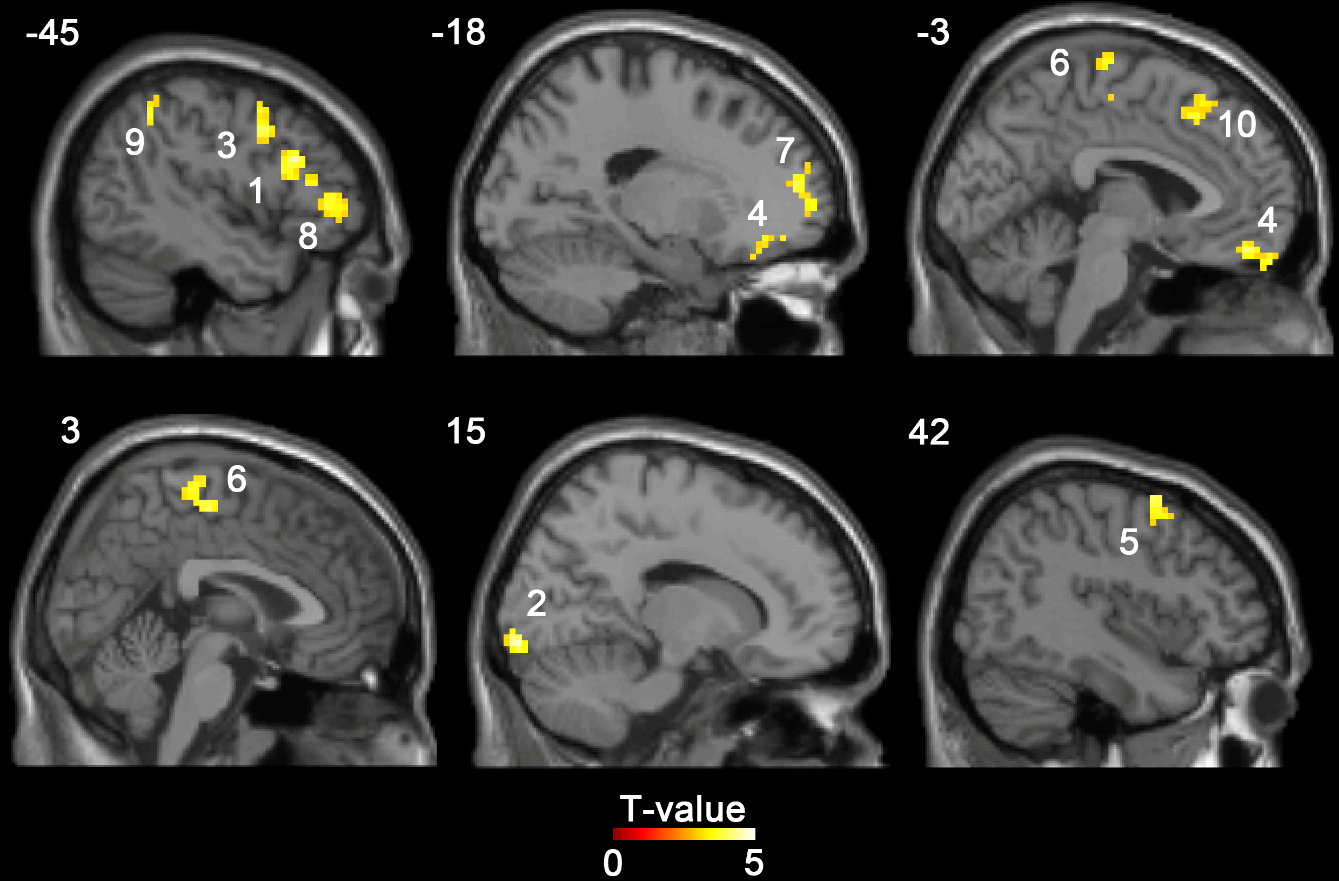


**Supplementary Table 8** – Description of the 1 significant cluster showing greater brain response to no loss (vs. loss) feedback in the young adults prenatally exposed to alcohol (independently of sex, maternal education and maternal depression during pregnancy).

| # cluster | Hedges’ g | cluster – level | | | | Number of voxels per AAL region |
| --- | --- | --- | --- | --- | --- | --- |
|  |  | p FWE | # voxels | T-value in maximum | x,y,z {mm} |  |
| 1 | 0.753 | 0.040 | 35 | 4.06 | 18 -97 -10 | 28 Lingual_R; 6 Calcarine_R |

**Supplementary Figure 7 –** Right lingual gyrus showed greater brain response to no loss (vs. loss) feedback in the young adults prenatally exposed to alcohol (independently of sex, maternal education and maternal depression during pregnancy).


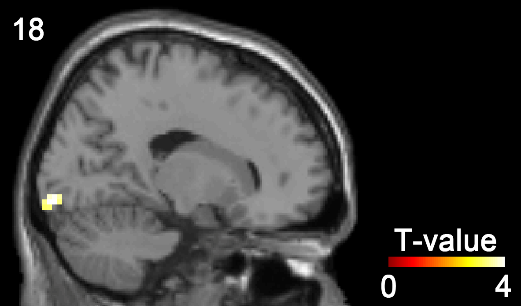

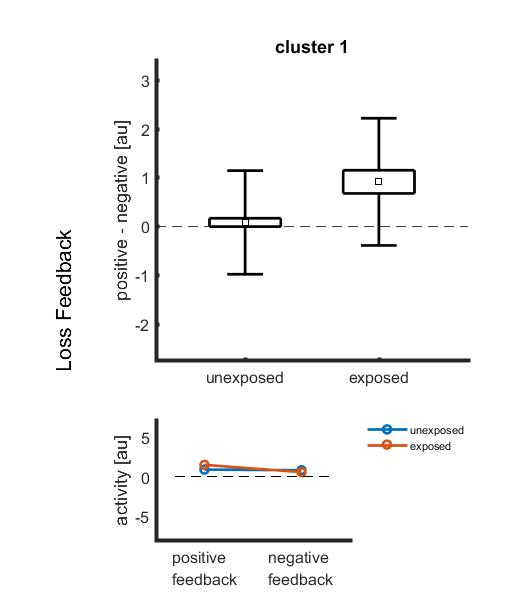


**Supplementary Table 9** – **Sex differences in the effects of alcohol exposure when correcting for maternal education and maternal depression during pregnancy.** 2A - Men exposed to alcohol in mid-pregnancy showed greater brain response to no loss (vs. loss) feedback in putamen, inferior temporal region, and occipital region.

| # cluster |  | cluster – level | | | | Number of voxels per AAL region |
| --- | --- | --- | --- | --- | --- | --- |
|  | ΔR^2^ | p FWE | # voxels | T-value in maximum | x,y,z {mm} |  |
| 1 | 0.123 | 0.003 | 59 | 4.88 | -27 -13 8 | 41 Putamen_L; 4 Pallidum_L |
| 2 | 0.111 | 0.020 | 41 | 4.62 | -57 -37 -22 | 41 Temporal_Inf_L |
| 3 | 0.103 | 0.020 | 41 | 4.17 | -21 -76 23 | 9 Occipital_Sup_L; 8 Occipital_Mid_L; 6 Cuneus_L; 4 Calcarine_L |

**Supplementary Figure 8 -** Men exposed to alcohol in mid-pregnancy showed greater brain response to no loss (vs. loss) feedback in putamen, inferior temporal region, and occipital region and these results were independent of maternal education and maternal depression during pregnancy.


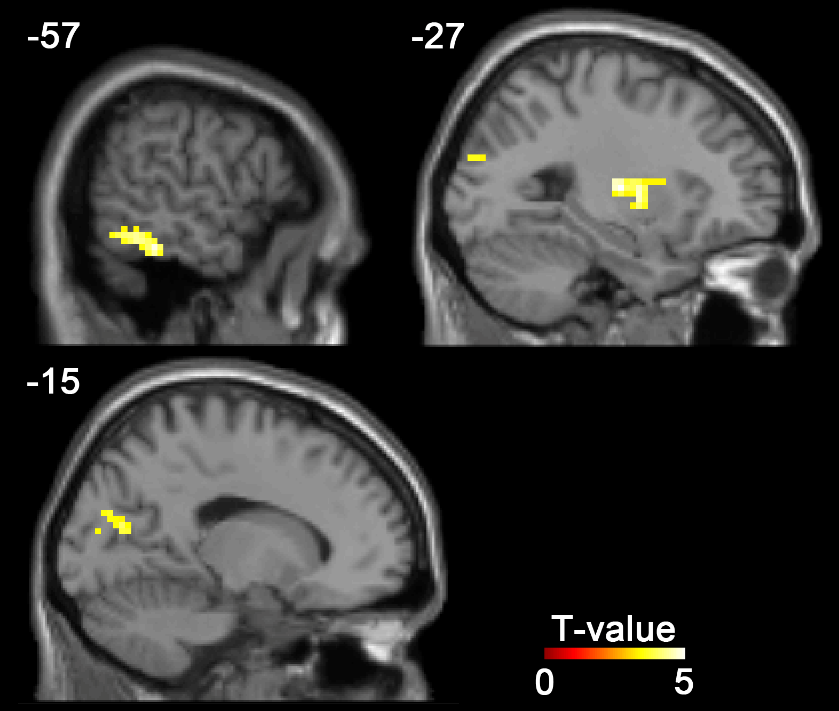


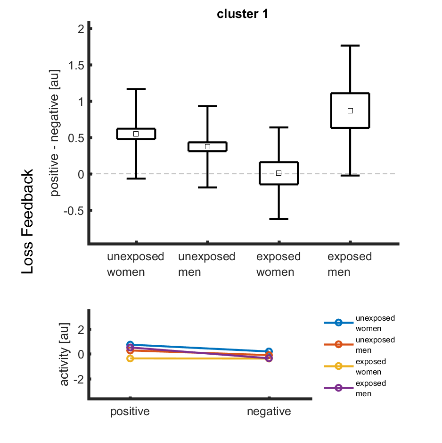

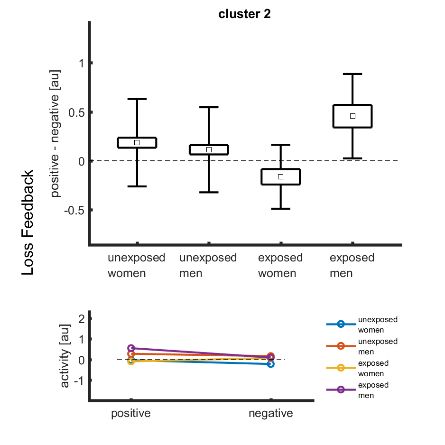

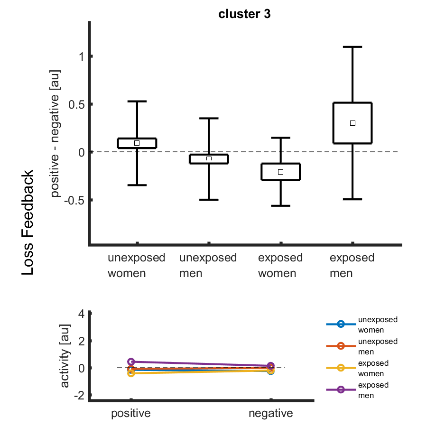

Supplement: Supplementary file 1 — Supplementary Material [file 41398_2024_2941_MOESM1_ESM.docx]
